# A socio-economic analysis of increased staffing in the Norwegian helicopter emergency medical service

**DOI:** 10.1186/s13049-018-0548-4

**Published:** 2018-09-21

**Authors:** Eirik Bjorheim Abrahamsen, Jon Tømmerås Selvik, Anders Nordgaard Dahle, Frank Asche, Håkon Bjorheim Abrahamsen

**Affiliations:** 1 Department of Safety, Economics and Planning, University of Stavanger, Stavanger, Norway; 2 Department of Flight Operations, Norwegian Air Ambulance, Oslo, Norway; 3 Institute for Sustainable Food Systems and School of Forest Resources and Conservation, University of Florida, Gainesville, Florida USA; 4 Department of Anaesthesiology and Intensive Care, Stavanger University Hospital, Stavanger, Norway

**Keywords:** Helicopter emergency medical service (HEMS), New regulation, Increased staffing, Socio-economic analysis

## Abstract

**Background:**

The European Aviation Safety Agency (EASA) is preparing a new set of regulations that will cover working and resting periods for crew members engaged in emergency medical services with helicopters (HEMS) and aeroplanes (AEMS). Such a shared European regulatory framework has already been introduced for the majority of commercial operations with aeroplanes, whereas national regulations are still in place for helicopter operations. A possible consequence of changing the regulations on working and resting periods for helicopter operations is that current abilities to provide 24-h, continuous emergency readiness with the same helicopter crew will be changed to a daily shift pattern with two, and even up to three, different crews to cover one 24-h period.

**Methods:**

A cost-benefit study is used to analyse whether changed working and resting periods, through the introduction of a shared European framework are socio-economically profitable for Norwegian helicopter emergency medical service (HEMS). For the study, relevant data is available for the total of nine HEMS helicopters of the three regions in Norway, for the period 2006–2013.

The aim of the study is to document whether changed working and resting periods will be socio-economically beneficial for the Norwegian HEMS.

**Results:**

The expected present value of changing the current regulations on working and resting periods is estimated at negative 181 million NOK over a 40-year period. This includes the assumption that all missions that are not completed today due to limitation in crew availability will be completed upon introducing new working and resting periods. In the current regulatory regime for the Norwegian HEMS, there are on average seven missions per HEMS base annually that are not completed due to the limitations in crew availability with the current working and resting periods. Changing the regulations on working and resting periods is estimated to be cost-effective when a minimum of 14 missions per year are prevented from being cancelled due to crew availability.

**Discussion:**

The benefit and cost elements used in the socio-economic analysis contain an estimated benefit of the measure, based on the valuation of life years gained for a limited number of patients. The prerequisites for life years gained, with the associated monetary value for quality-adjusted life years, are important for the outcome of the cost-benefit analysis. In this study 6.95 life years gained is used as basis for the benefit of the measure. This number is based on the conclusion of two studies, which have studied the benefits of HEMS helicopters staffed with a doctor in Norway.

In a cost-benefit analyses, a quantification shall as far as possible be made in monetary values of all the positive and negative effects the measure entails. In this analysis, one criticism may be that these effects are relatively few, the investment costs (the increased operating costs) are not provided a detailed description of, and other factors such as; effect on the environment, risk of simultaneous requirements of the HEMS helicopter with possible negative effect for the patient who most needs it, likelihood of accidents with associated negative effect are neither included in the cost-benefit analysis.

**Conclusion:**

Alternations to the working and resting periods for Norwegian HEMS operations that will result in a change from the current 24-h, continuous emergency readiness with the same crew, to a set-up with two, and up to three, different crews are not found to be socio-economically beneficial.

## Background

In Norway, a health policy goal is to ensure that equal healthcare is offered to the entire population, regardless of where they live (geographical justice), their age, sex and social status. This includes similar access to both emergency medical services and specialised treatment services regardless of place of residence [1]. As in many other countries, HEMS (helicopter emergency medical service) helicopters are essential for providing these services in remote areas. The main tasks of the HEMS helicopters are to offer advanced emergency medical treatment outside hospitals and to bring patients directly to the correct level of treatment in the health service. However, it is increasingly costly to provide equal services to the most remote areas, and trade-offs are unavoidable. The central guiding document is the Official Norwegian Report [2], recommends that 90% of the population should be reached by an ambulance with a doctor within 45 min.

The medical benefits of HEMS are primarily related to emergency medical competence and the time factor. Emergency medical expertise is ensured by having doctors and nurses with a specialisation in emergency medicine as crew members. The time factor covers both how long it takes from when the emergency medical condition occurs until adequate emergency care is given, and the time taken from when the condition occurs until the patient reaches hospital for treatment [1]. Conditions such as weather, simultaneous emergency requirements, patient-related conditions and down-time due to technical issues or crew availability are factors that may prevent an HEMS helicopter from completing a desired mission. Crew availability is limited due to regulations that control the number of cumulative or continuous active hours. Once the hour limit is reached, the crew on the helicopter must be taken out of service to rest before a new active service can commence.

For the majority of European commercial operations with aeroplanes, there is a shared European regulatory framework covering working and resting periods for crew members. Such a framework is, however, not available for helicopter operations, although The European Aviation Safety Agency (EASA) is working on a set of regulations on this issue. The forthcoming new framework could challenge the current continuous emergency readiness (24 h) using the same helicopter crew and could influence the quality of the emergency response. This paper studies how the suggested limitation in available service hours affects the completion of the desired missions in the current system. The introduction of common European regulations for working and resting periods may lead to a reduction in down-time due to better crew availability, but it will also involve increased costs due to investment in more crews and helicopters. Based on this, we investigate whether the cost of investing in increased staffing of the state HEMS helicopters, to avoid missions not being carried out due to limitations in crew availability, can be justified by the benefits associated with carrying out these missions.

## The Norwegian HEMS

The Norwegian HEMS are organised into 12 HEMS bases and 13 helicopters on standby 24 h a day, all year round. A regulation from the Ministry of Transport and Communications Norway [3], i.e. the ‘BSL D 2-4’, governs the staffing arrangements of the HEMS helicopters, including allowing 24 h of continuous emergency readiness service for Norwegian HEMS crews. Furthermore, according to the ‘BSL D 2-4’ regulations, emergency readiness on land (on a base) shall, as a minimum count as 50% of the active working hours. This means that 24 h of emergency readiness with no actual duty will count as 12 h of working time against a 12-month limit of 2000 h. For actual duty, such as service on board an aircraft with associated preparation and post-flight duties, simulator training, other training or any other administrative duties, the time shall be counted as full working time and be deducted from the time calculated as emergency readiness service. In practice, this means that the actual working hours of crew members engaged in HEMS are calculated in arrears, as it is not possible to predict exactly the daily workload.

In order to ensure a sufficient buffer for unforeseen circumstances within the annual limit of 2000 h of working time, the number of crews is calculated based on a normal man-year of 1750–1900 h, which gives a normal shift pattern of approximately 104 shift days plus days for the completion of necessary operational training. The minimum staffing requirement is four crews per HEMS helicopter. The tactical disposition of working hours while the employee is actually at work is regulated according to the individual operator’s operating manual. Today, there is no shared regulatory framework that the individual operators must follow in the design of such manuals.

In 2008, new working and resting periods were introduced for crew members on aeroplanes, including ambulance aeroplanes. One consequence was 24-h shifts became illegal, creating a need for two crews on each aircraft to cover a whole day. As a result, the number of pilots involved in the ambulance operation had to be increased by almost 90% [4]. The cost increase for the ambulance operations was primarily related to increased personnel costs, as it was not necessary to increase the total number of flight hours in order to ensure an average minimum number of flight hours per pilot (to ensure minimum level of proficiency). The average went from approximately 400 to 250 flight hours per year per pilot [4].

If the helicopter regulations are similar to the airplane regulations, the National Air Ambulance Services of Norway estimates that the number of crew per HEMS helicopter on 24 h readiness service will increase from 3.5 to 5. In order to safeguard the limitations in working hours which would result from such new regulations, a need for additional back-up crews is also expected. HEMS helicopters crews in Norway currently average approximately 200 flight hours per year, most of which are based on real ambulance missions. In the Norwegian Air Force, less than 180 flight hours per year is not recommended, in order to maintain an acceptable level of proficiency, which is a recommendation that the National Air Ambulance Services of Norway supports [4]. If such regulations were introduced, the HEMS helicopter operation would also have to increase its number of flight hours to continue to maintain an acceptable level of proficiency for helicopter crews. In practice, this means that the number of helicopters must be increased by 50% and the number of flight hours by 70% [4].

## Method

To investigate whether the new regulations are socio-economically beneficial, a cost-benefit analysis is conducted. In this analysis, we calculate the expected net present value, E[NPV], which provides a weighted average of the estimated costs and benefits associated with an investment [5]. The NPV is calculated using the annual benefits B_t_ and the costs C_t_ as:

$$ NPV=\sum \limits_{t=0}^T\frac{B_t-{C}_t}{{\left(1+{r}_t\right)}^t}, $$

Here, T is here the time period considered (in years) and r is the required rate of return, or discount rate, at year t. The values are estimated using expected values as the cash flows are future values, and the discount rate is set at 4% following the recommendation by the Norwegian Ministry of Finance for socio-economic analysis [6].

Below, we will discuss how the key parameters, necessary to compute the expected net present value of the new regulations for emergency helicopter services are obtained. The values are summarised in Table 1. Benefits and costs will be computed in Norwegian kroner (NOK). Where a business economic analysis uses market prices in the valuation of different measures, it is necessary to use calculation prices in socio-economic analyses, as market values are not available for the benefits associated with completing more helicopter missions. The resources associated with the additional crews, or the input factors, are calculated at market prices.

**Table 1:** Application of theory for conducting socio-economic analysis

What	Number
Calculation rate	4%
Analysis period	40 years
Benefit	92.80%
Quality-Adjusted Life Years	1.111 mill

In the implementation of socio-economic analyses, we follow common practice and allow real prices for a project’s benefits and costs to be constant throughout the analysis period. This is based on an assumption that all benefits and costs have the same price trend as the consumer price index [6].

The Directorate of Health assesses that the value of 1 QALY (Quality-Adjusted Life Years; see below section on selection of a measurement for assessing the benefits of a health measure) may vary from 588,000 to 1120 million 2012-kroner [7]. We use the highest value of 1 QALY set to 1120 million 2012-kroner and adjusted it with the consumer price index to get the value in 2011-kroner. It is important to have the value in 2011-kroner since, in their analysis of the consequences for a change in the work and rest regulations, the National Air Ambulance Services of Norway estimated an increase of 221 million kroner in the annual helicopter operating cost of the air ambulance service [4].

In order to determine the scope of the analysis, one must define the lifetime of the measure. Lifetime is defined as the period in which the measure to be analysed is to be used or in which it will provide a societal service. A time period of 40 years is used in our analysis regarding the recommendations from The Ministry of Finance Norway [6].

To compute benefits when evaluating various health measures, an estimate of Life Years Gained (LYG) or Quality-Adjusted Life Years (QALYs) are used. The main difference between them is that life years gained only measures mortality, while quality-adjusted life years seeks to include the effects of disease on the quality of life years. The similarity is that both measurements add more weight to the younger over the older, compared with a single mortality measurement of avoided deaths [8].

If benefit is expressed in terms of life years gained, it is necessary to adjust this to quality-adjusted life years, since there is a recommended financial value in kroner for a quality-adjusted life year. The report, “Gained Quality-Adjusted Life Years (QALYs) with Physical Activity” [9], included research on the level of health benefits in terms of life years gained and quality-adjusted life years gained that is correct to use in socio-economic analyses of preventive measures that increase physical activity. Based on [9], it is derived from the factors that are researched in the report that the average percentage increase from life years gained to quality-adjusted life years gained is 92.80%.

The Directorate of Health recommends in its guidelines on “Economic Evaluation of Health Measures” [10] using half a million 2005-kroner as the authorities’ reference value for *a statistical life year of full-health* (quality-adjusted life year, 1 QALY) as a temporary value. In a note of January 2014, the Directorate of Health [7] discusses what can be used as the sectoral value of a quality-adjusted life year (1 QALY). Depending on the statistical value of a life one wants the quality-adjusted life year value to be consistent with, the Directorate of Health assesses that the value of 1 QALY may vary from 588,000 to 1120 million 2012-kroner [7]. We chose the maximum value of 1120 million kroner and adjust it to the 2011 value using the CPI.

## Results

The benefits of changing the working and resting regulations will be related to fewer cancelled missions. When introducing new regulations, it is assumed that no missions will be cancelled due to improved crew availability due to the increased staffing. While this is unrealistic, as there almost certainly will be cancellations. However, this is difficult to estimate based on historical data. When assuming zero cancellations, out benefit estimate may therefore be biased upwards.

**Fig. 1:**
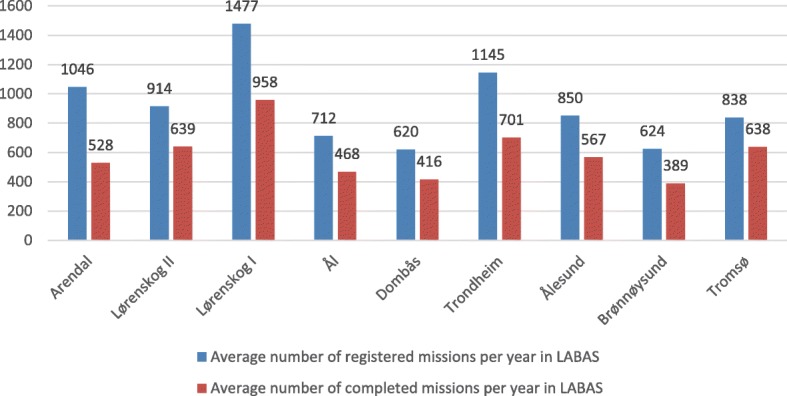
Average number of registered missions per year in LABAS (blue columns) and the average number of completed missions per year in LABAS (red columns) during the period 2006 to 2013 for the HEMS helicopters belonging to Helse Nord, Helse Midt-Norge and Helse Sør-Øst

During the period 2006 to 2013, a total of 65,802 missions were registered in the database LABAS for the nine HEMS helicopters associated with the health regions Helse Nord, Helse Midt-Norge and Helse Sør-Øst on the HEMS bases in Tromsø, Brønnøysund, Ålesund, Trondheim, Dombås, Ål, Lørenskog and Arendal. Of these, 42,428 missions were completed. The activity data is shown in Fig. 1. Activity data for the individual years is not presented, only the average number of missions and completed missions in the period 2006 to 2013.

**Fig. 2:**
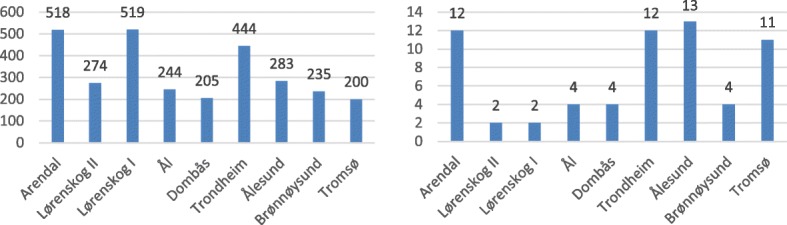
Average number of cancelled missions (left side diagram) and average number of missions cancelled due to crew availability (right side diagram) in the period 2006 to 2013 for HEMS helicopters belonging to Helse Nord, Helse Midt-Norge and Helse Sør-Øst

In the period 2006 to 2013, annually an average of 325 registered missions were not carried out for various reasons [technical problems, patient-related conditions (no longer required, patient not transportable), simultaneous requirements, crew availability and weather]. The variation in the distribution is a maximum average for the HEMS helicopter in Lørenskog (Lørenskog helicopter) with 519 missions and a minimum average for the HEMS helicopter in Tromsø with 200 missions. The variation is 319 missions. See Fig. 2.

During the same period, an annual average of seven registered missions were not completed due to crew availability, where the variation in the distribution is 11, i.e. the difference between the maximum average (the HEMS helicopter in Ålesund with 13 missions) and the minimum average (the HEMS helicopters “Lørenskog I” and “Lørenskog II” both have two missions). See Fig. 2b*.*

Registered activity data shows variations between the different HEMS bases with regard to share of missions that were not completed due to crew availability. At the Lørenskog base, there is a high number of completed missions, and the proportion of missions ‘not completed due to *crew availability*’ is low. Statistics for the HEMS helicopter in Tromsø are close to the average, in terms of both requested mission and completed missions, while the share of registered missions ‘not completed due to *crew availability*’ is higher than average. This is likely to be due to two factors. One can be different practices for registering requested missions for the HEMS helicopter when it has *already* been taken out of service due to *crew availability* or *technological issues*. At the University Hospital of North Norway, there has been an established practice for several years that all requested missions for the HEMS helicopter are registered – regardless. The second may be that, until 2014, there were different guidelines for registering active time with the corresponding need for rest between, at the time, the two HEMS helicopter operators in Norway. This may have resulted in one operator (Lufttransport AS) having less available active service time and, thus, a higher number of unsuccessful missions due to *crew availability*. As there are no shared provisions for working and resting periods for helicopter operations, the individual operator must establish guidelines for this and get them approved by the Civil Aviation Authority as part of their operating manual. The change which Lufttransport AS undertook in 2014 has resulted in a noticeable improvement in the availability of HEMS helicopters, which has increased from about 95% in 2013 to about 97% in 2014 [11].

Based on the inputs from Table 1, a net present value calculation has been made for the introduction of a shared European regulatory framework for the Norwegian HEMS. The results are reported in Table 2. The Average LYG in Table 2 is set equal to 6.95 based on the studies [12, 13].

**Table 2:** Calculation of value for quality-adjusted life years for the missions

Patients			Life Years Gained (LYG) calculated as Quality-Adjusted Life Years (QALY)				Value of Quality-Adjusted Life Years (QALY)		
[Number of missions]	[Percent of missions where patient benefits in terms of LYG]	[Number LYG]	[Average LYG]	[Increase LYG- > QALY]	[Number]	[Average QALY]	[Number QALY]	[Value QALY]	[Number; Net Benefit]
7	8.89%	0.62	6.95	92.80%	6.45	13.40	8.33	1,111,476	9,262,955

As one can see, the introduction of the measure limiting flight hours will result in seven requested missions (which were not previously completed due to crew availability) being carried out. However, as the cost of maintaining the additional crews is much higher than the benefits associated with completing these missions, the net present value is a negative 181 million NOK.

**Fig. 3:**
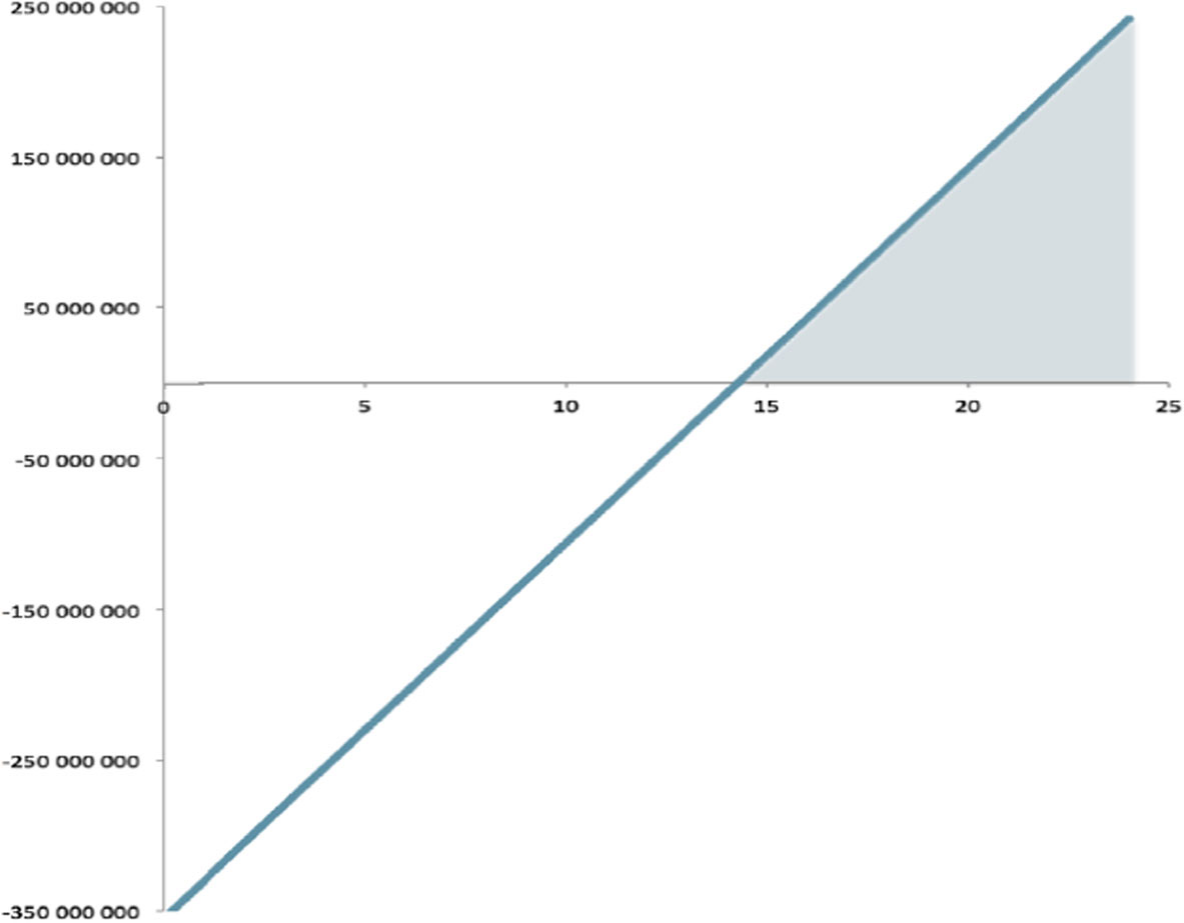
Sensitivity assessment on how the number of missions (x-axis) affects the net present value in NOK (y-axis)

There are several uncertainties connected with this cost-benefit analysis. One of the more important of these is whether or not the set prerequisites are reasonable. However, a sensitivity analysis can shed light on the robustness of the result. It shows how the effect of changes to the number of missions, where we are able to compute how many missions must be performed to give a positive net present value. In Fig. 3, the net present value is calculated for a mission range of 0 to 24 showing the sensitivity of the net present value results. The graph is linear, since both the benefit and the cost of the measure are assumed to be constant and to occur simultaneously during the analysis period of 40 years.

The measure gives a positive net present value from 15 missions (rounded). The area in the figure where the number of missions give a positive net value is marked with grey colour. As mentioned earlier, it is likely that there may be an error source in the data with regard to how many requested missions were not completed due to crew availability. For the HEMS helicopter in Tromsø, such missions account for 1.27%, which is 0.50% higher than the average (0.77%) for the nine helicopters for which activity data exists. The average number of annual missions (638) for the HEMS helicopter in Tromsø is above the average number of missions (589) for the nine HEMS helicopters in the period from 2006 to 2013. Assuming that 1.27% reflects a more correct estimate of ‘missions not completed due to crew availability’, such a percentage would mean that, 12 missions are not completed for this reason by the HEMS helicopter.

If 12 is a more correct figure for the average number of missions ‘not completed due to crew availability’, a net present value of the measure will be negative 56.7 million Norwegian kroner. The benefit of the measure presented in life years gained is based on an average of two medical studies conducted in Norway [12, 13]. Both studies are relatively old, considering the developments in acute medicine and medical devices in the last decade. A study conducted in Denmark [14] provides the basis for an assumption that the benefit value of HEMS helicopters staffed with a doctor is higher for society than previous research indicates. In this study, there were 204 patients (56 before the introduction of HEMS helicopters staffed with a doctor, and 148 patients after) from a population of 1788 who were considered to be seriously injured, representing 11% of patients. For the severely injured, 30-day mortality was reduced from 29% before the introduction of a HEMS helicopter to 14% the following year. There was also a significant reduction in mortality among those with a lower level of injury in the post-establishment period. If all other conditions are assumed to be the same and only the benefit in number of life years gained increases, then an average of 14.18 life years gained will give a positive net present value for the measure. However, as noted above, zero cancellations due to crew ability is unrealistic even with the new regulations. If these were accounted for, the distance to the break-even would increase again.

## Discussion

The benefit and cost elements used in the socio-economic analysis contain an estimated benefit of the measure, based on the valuation of life years gained for a limited number of patients. In addition, investment costs are based on an estimate made by the National Air Ambulance Service of Norway, averaged out to associate them with a ‘statistical HEMS helicopter’.^1^ The reliability of the analysis is of course dependent on the quality of these estimates.

Available activity data is used to calculate i) the average number of requested missions, ii) the average number of completed missions and iii) the average number of not completed missions. Such averages do not necessarily represent any of the actual HEMS helicopters bases in terms of their activity profile. For some of the HEMS helicopter bases the activity level is much higher than average, and for some the activity level is lower. As mentioned earlier, there may be a source of error in how many requested missions are registered as ‘not completed due to crew availability’ and the actual number of requested missions that are received when the crew is out of service. The HEMS helicopter service in Tromsø states that all requested missions are registered and logged. Thus, the average number of 12 missions per year ‘not completed due to crew availability’ is considered reliable. At the same time, in 2014, the National Air Ambulance Service of Norway registered an increase in availability of approximately 2 % after Lufttransport AS introduced new provisions for accumulated/continuous active service and resting periods. This may mean that the annual average number of missions not completed due to crew availability is reduced. Nevertheless, based on the chosen prerequisites, relatively few missions are needed before the new regulations for working and resting periods are considered socio-economically profitable.

The prerequisites for life years gained, with the associated monetary value for quality-adjusted life years, are important for the outcome of the cost-benefit analysis. Life years gained will depend on several conditions: among other things, demographic data in the population will indicate an average expected remaining lifetime. Population development in Norway has been in the direction of stronger population growth in urban areas and larger urban settlements where the settlement pattern now shows a clear concentration in relatively small areas. This is mainly in the central eastern region, as well as in concentrations along the coast. In northern Norway in particular 90% of the population lives less than four kilometres from the sea with the densest concentration of settlements along the coast of Helgeland, in Lofoten and Vesterålen, and along the inner coastal waters in Sør-Troms from the county border from Nordland to Tromsø [15]. In addition, a larger proportion of older people is expected in the future population [16]. Together, these factors indicate an increase in urbanisation, combined with a higher proportion of elderly people in the population with a lower average expected remaining lifetime.

HEMS helicopters are mainly used for missions in rural areas, where their use gives benefits in terms of time and emergency medical competence, meaning that the assumption of an average of 6.95 life years gained can be representative. At the same time, this is a lower average than the number of life years lost to cancer in Norway (13.8 years [17]), the average expected remaining lifetime for both sexes which in 2011 was 43.07 years in Norway, and the findings of a study [14], which indicates a 15% reduction in 30-day mortality for severely injured patients following the introduction of HEMS helicopters staffed with a doctor. In one [12] of two medical studies, 96% of the 290.6 life years gained (for patients considered to have benefited from the service) was achieved amongst nine patients, six of whom were under seven years old, while four were aged between 0 to 7 months. The overall assessment is that the prerequisite for using 6.95 life years gained in the cost-benefit analysis can be defended.

In cost-benefit analyses, a quantification shall as far as possible be made in monetary values of all the positive and negative effects the measure entails. In this analysis, one criticism may be that these effects are relatively few. In terms of investment costs (the increased operating costs), it does not provide a detailed description of what they include, which makes it difficult to test/analyse them.

To provide a better basis for decision-making, the cost-benefit analysis could have included a thorough calculation in monetary values of all the positive and negative effects that the implementation of a new European framework for working and resting periods would entail. Some factors that may affect the result are:

### Environment

It is reasonable to assume that the introduction of new regulations will increase the number of flight hours due to more accepted missions, since the available service period will be greater. Either way, this is included in the higher operating costs associated with the need for more flight hours to maintain proficiency and qualification standards. The increase in flight hours will be a negative effect of the measure due to the impact it will have on the environment in terms of increased air and noise pollution.

### Simultaneous requirements

HEMS helicopters staffed with an anaesthesiologist constitute a specialist health service for bringing emergency medical assistance to severely injured and ill patients. The measure will lead to greater accessibility in the HEMS helicopter service, because in theory it will no longer be possible to opt out of a mission due to service hour restrictions. If such increased accessibility would lead to a lower threshold for accepting missions, it is reasonable to assume that it will give a greater likelihood of simultaneous requirements occurring. This can at worst lead to time-critical assistance not arriving in time for the patient who most needs it, and any possible increase in resource conflicts due to the introduction of the measure will be a negative effect.

### Accidents

The likelihood of accidents will have a very negative effect in a cost-benefit analysis. The purpose of changes to the working and resting periods for HEMS operations in Europe is to increase aviation safety by ensuring that HEMS crew members are at all times rested and ready to perform helicopter operations. At the same time, a possible consequence of such a change in regulation is a lowering of proficiency by reducing the number of flight hours to below what is accepted as a safe level (by the National Air Ambulance Service of Norway set to 160 h minimum in the new contract which started in June of 2018).

A more thorough analysis should be carried out of what the changes to the working and resting periods for HEMS operations in Norway would mean for safety and whether the likelihood of accidents will be increased, reduced or expected to remain at the same level as that for the current regulation. The outcome of such an analysis will have a big impact on how socio-economically profitable the option will be.

### Option value

The measure is assumed to provide higher availability of the HEMS helicopters, since cancellations due to crew availability will no longer be a factor. Option value is applied to changes in accessibility that are not necessarily reflected in increased use of a means of transportation [18]. For the general public, it is reasonable to estimate that the measure will result in an increase in option value – and thus give a positive effect – by increasing the availability of the HEMS helicopter.

### Input for future work

The analysis is performed using the available and relevant data covering the full period 2006 to 2013. However, if the EASA are delayed in their schedule to develop regulations applicable to HEMS operations, a more recent data set should be collected to analyse updated socio-economic effects. Furthermore, if the EASA implements the regulations currently under development on working and resting periods for crew members in helicopter operations, the effects under the new operating conditions should also be studied.

## Conclusion

The current organisation of the HEMS helicopter operation makes it possible to offer 24-h emergency readiness with the same crew. During the period 2006 to 2013, this has meant that 0.75% of the total number of missions (*n*  = 65,802) for nine HEMS helicopters has been registered as ‘not completed due to *crew availability*’. For the *statistical* HEMS helicopter, this means that on average seven missions per year have not be completed for this reason. The possible introduction of new regulations for working and resting periods that will result in a shift pattern with two crews is assumed to mean they will complete the missions that are not currently completed due to crew availability.

Socio-economic analyses are used to map, systematise and visualise information that is relevant for facilitating the decision-making of whether or not a certain measure should be introduced. The possible introduction of the proposed measure as described in this paper is not decided by the Norwegian Aviation Authorities, Health Authorities or the Norwegian Air Ambulance Service. In the current base option, the average number of flight hours per crew is just over 180 flight hours per year, which is the minimum number of flight hours recommended to maintain a safe level of proficiency. The proposed measure is expected to reduce the average flight time per crew to such an extent that it is likely to negatively affect flight safety – even if the objective is to increase it. Given the low number of requested missions that are currently not completed due to *crew availability*, it seems irresponsible to possibly increase the cost to such an extent for something that would have a negative benefit, while at the same time threatening both the capacity and structure of the entire HEMS helicopter section of the air ambulance service.

The benefit value of HEMS helicopters staffed with an anaesthesiologist is expressed as life years gained in the medical studies that have examined the health effect for those patients who underwent treatment by the anaesthesiologist who is part of the HEMS helicopter staff. By using these studies as an empirical asset for the cost-benefit analysis of the measure, the analysis shows a negative net present value of 181 million Norwegian kroner. The investments that will have to be made in crews and helicopters to implement the measure exceed what society receives in terms of saved quality-adjusted life years. The conclusion of the research question is therefore that it would be economically unprofitable to introduce the measure: thus, its introduction is not recommended.

## Abbreviations

CAPM: Capital Asset Pricing Model
EASA: the European Aviation Safety Agency
HEMS: Helicopter Emergency Medical Service
LYG: Life Years Gained
QALY: Quality-Adjusted Life Years

## Footnotes

1 The statistical HEMS helicopter is the average of the 9 HEMS helicopters of the three regions in Norway. The relevant data available is for the period 2006–2013.
